# Genetic variants of ADAM9 as potential predictors for biochemical recurrence in prostate cancer patients after receiving a radical prostatectomy

**DOI:** 10.7150/ijms.103179

**Published:** 2024-11-04

**Authors:** Yung-Wei Lin, Yu-Ching Wen, Chia- Yen Lin, Chi-Hao Hsiao, Kuo-Hao Ho, Hsiang-Ching Huang, Lun-Ching Chang, Shian-Shiang Wang, Shun-Fa Yang, Ming-Hsien Chien

**Affiliations:** 1 International Master/PhD Program in Medicine, College of Medicine, Taipei Medical University, Taipei, Taiwan.; 2 Department of Urology, School of Medicine, College of Medicine, Taipei Medical University, Taipei, Taiwan.; 3 Department of Urology, Wan Fang Hospital, Taipei Medical University, Taipei, Taiwan.; 4 Division of Urology, Department of Surgery, Taichung Veterans General Hospital, Taichung, Taiwan.; 5 School of Medicine, Chung Shan Medical University, Taichung, Taiwan.; 6 School of Medicine, National Yang Ming Chiao Tung University, Taipei, Taiwan.; 7 Graduate Institute of Clinical Medicine, College of Medicine, Taipei Medical University, Taipei, Taiwan.; 8 Department of Mathematical Sciences, Florida Atlantic University, Boca Raton, FL, USA.; 9 Department of Applied Chemistry, National Chi Nan University, Nantou, Taiwan.; 10 Institute of Medicine, Chung Shan Medical University, Taichung, Taiwan.; 11 Department of Medical Research, Chung Shan Medical University Hospital, Taichung, Taiwan.; 12 Pulmonary Research Center, Wan Fang Hospital, Taipei Medical University, Taipei, Taiwan.; 13 Traditional Herbal Medicine Research Center, Taipei Medical University Hospital, Taipei, Taiwan.; 14 TMU Research Center of Cancer Translational Medicine, Taipei Medical University, Taipei, Taiwan.

**Keywords:** A disintegrin and metalloprotease 9, Single-nucleotide polymorphism, Biochemical recurrence, Clinicopathologic progression, Prostate cancer

## Abstract

A disintegrin and metalloproteinase domain-containing protein 9 (ADAM9) functions as a membranous bridge, forming cell-cell and cell-matrix connections that regulate tumor aggressiveness in various cancer types, including prostate cancer (PCa). Elevated ADAM9 levels in PCa were identified as a prognostic marker for biochemical recurrence (BCR) in patients who had undergone a radical prostatectomy (RP). However, impacts of genetic variants of ADAM9 on clinicopathological development and BCR remain unclear. Herein, we recruited 702 patients with PCa to evaluate associations of single-nucleotide polymorphisms (SNPs) of ADAM9 with the risk of BCR and clinicopathological development. We genotyped four loci of ADAM9 SNPs located in the promoter and intron regions using a TaqMan allelic discrimination assay, including rs10105311 (C/T), rs7006414 (T/C), rs6474526 (T/G), and rs78451751 (T/C) in 702 Taiwanese PCa patients. Our results showed that the risk of postoperative BCR was 1.508-fold higher in patients carrying the T/C genotype in ADAM9 rs7006414 compared to those with the homozygous T/T genotype, a phenomenon more pronounced in younger PCa patients (aged ≤ 65 years). Furthermore, patients with at least one polymorphic G allele in ADAM9 rs6474526 had a 2.016-fold increased risk of developing an advanced clinical primary tumor stage, particularly in a subpopulation without BCR. Clinical observations from the Genotype-Tissue Expression (GTEx) database showed increased ADAM9 expression in whole blood tissues among individuals carrying the polymorphic C allele of rs7006414 and the G allele of rs6474526. Additionally, data from The Cancer Genome Atlas indicated that elevated ADAM9 levels were observed in PCa tissues compared to corresponding matched normal tissues. Our findings suggest that the rs7006414 and rs6474526 genetic variants of ADAM9 may influence ADAM9 expression and are associated with BCR and clinicopathological development in PCa patients after an RP.

## Introduction

Prostate cancer (PCa) is the second leading cause of cancer-related mortality in males, particularly in Western countries 1. Recently, there has been increasing trends in PCa incidences in developed countries, including Taiwan, likely due to more-advanced medical care facilities and early-stage prostate-specific antigen (PSA) screening 2. While early-stage PCa can be effectively managed through a radical prostatectomy (RP) and radiotherapy, 20%-50% of PCa patients will experience biochemical recurrence (BCR) within 10 years after initial definitive therapy, characterized by rising serum PSA levels 3. Although PSA is used as a biomarker for PCa screening, detection, and postoperative tumor recurrence diagnosis, its accuracy is limited. For instance, elevated serum PSA levels can occur in PCa and also in other non-malignant conditions such as prostatic hyperplasia, prostatitis, an older age, and an increased prostate volume 4. Moreover, the PSA threshold indicative of BCR after an RP has long been debated, with no consensus on the best PSA threshold for defining BCR 5-7. Therefore, given the importance of early PCa diagnoses for preventing progression and facilitating timely treatment, it is crucial to conduct research aimed at identifying novel and efficient predictive biomarkers.

Recent epidemiological studies suggested that PCa is a highly heritable malignancy, indicating a strong causal association between genetic factors and PCa development 8. Single-nucleotide polymorphisms (SNPs), which are variations in a genome's base pairs in DNA sequences, cause variations in genes that alter the protein and enzymatic machinery of cells 9,10. Genome-wide association studies and fine-mapping efforts have identified more than 100 well-recognized SNPs associated with PCa, which constitute major risk factors for susceptibility to, development of, and BCR of PCa 11,12. For instance, SNPs (rs12422149, rs1789693, and rs1077858) in androgen transporter genes, such as solute carrier organic anion transporter family member 2B1, may serve as pharmacogenomic determinants of resistance to androgen deprivation therapy (ADT) in PCa 13. Y-box-binding protein-1, a critical regulator of androgen receptor variants involved in resistance to ADT for PCa, has an intronic SNP (rs1203072) that affects gene expression and is linked to PCa metastasis 14. Additionally, a genetic interaction between insulin-like growth factor 1 (IGF1) rs2946834 and IGF1 receptor (IGF1R) rs2016347 was reported to be a predictor of BCR in PCa patients after an RP 15. Furthermore, the rs10895304 polymorphism of matrix metalloproteinase-7 is associated with an increased risk of BCR in PCa patients who have undergone an RP 16.

A disintegrin and metalloproteinase 9 (ADAM9) is a type I transmembrane glycoprotein and a significant member of the ADAM family. Recent studies demonstrated a relationship between elevated ADAM9 levels and progression of various cancer cells 17,18. ADAM9 is overexpressed in PCa and was reported to promote the transition from castration-sensitive PCa to castration-resistant PCa (CRPC) 19. Elevated ADAM9 was also identified as an independent prognostic marker for PSA relapse-free survival following an RP 20. Functionally, ADAM9 was reported to promote growth, metastasis, and therapeutic resistance in PCa through various mechanisms. For example, Tang *et al.* indicated that the ADAM9/WNT1-inducible signaling pathway protein 1 axis cooperates with osteoblasts to induce PCa growth and metastasis 21. Lin *et al.* reported that N-α-acetyltransferase 10 stabilizes ADAM9 to promote metastasis in CRPC 22. Additionally, Josson *et al.* showed that ADAM9 enhances radio- and chemoresistance in PCa by altering E-cadherin and integrin expressions 23. Although several studies investigated the clinical significance and functional roles of ADAM9 in PCa, impacts of genetic variants of ADAM9 on PCa have not been explored. In this study, we aimed to examine associations of SNPs within the *ADAM9* gene with the risk of BCR and clinicopathological development in Taiwanese PCa patients who had received an RP.

## Materials and Methods

### Study populations and ethics

In this study, we collected blood samples from 702 PCa patients who had undergone a robotic-assisted laparoscopic RP at Taichung Veterans General Hospital (Taichung, Taiwan) between 2012 and 2018. Prior to venous blood collection, written informed consent was obtained from each participant, and the study protocol was approved by the Institutional Review Board (IRB no. CE19062A-2) of Taichung Veterans General Hospital. Medical data for the recruited patients, including PSA values, pathologic Gleason grades, clinical and pathologic T (tumor) and N (node) staging, cancer cell invasion areas (perineural and lymphovascular regions), and D'Amico classification, were extracted from their medical records at the time of diagnosis. In our study, BCR among the recruited PCa patients was defined by the detection of two consecutive PSA measurements, each exceeding a threshold of 0.2 ng/mL. This criterion was used to indicate a potential recurrence of cancer following initial treatment.

### Genomic DNA extraction

Whole-blood samples from PCa patients were collected in tubes containing ethylenediaminetetraacetic acid. After centrifugation, genomic DNA was extracted from buffy coats using a QIAamp DNA Blood Mini Kit (Qiagen, Valencia, CA, USA) following the manufacturer's protocol. The quality of the isolated DNA was evaluated by a Nanodrop-2000 spectrophotometer (Thermo Scientific, Waltham, MA, USA) before it was used as a template for a polymerase chain reaction (PCR).

### Genotyping of ADAM9 SNPs

In this study, we selected four SNPs in the *ADAM9* gene: two in the promoter region (rs7006414 and rs10105311) and two in the intron region (rs6474526 and rs78451751). Alleles of ADAM9 SNPs of rs7006414 (assay ID: C_28949418_10), rs10105311 (assay ID: C_26007162_10), rs6474526 (assay ID: C_449039_10), and rs78451751 (assay ID: C_105349195_10) were discriminated using a TaqMan SNP Genotyping Assay on an ABI StepOnePlus™ Real-Time PCR System (Applied Biosystems, Foster City, CA, USA). Final results were analyzed with SDS vers. 3.0 software (Applied Biosystems). Detailed procedures of DNA genotyping were outlined in our previous study 24.

### Bioinformatics analysis

Clinical data and messenger (m)RNA sequencing for prostate adenocarcinoma (PRAD) samples from The Cancer Genome Atlas (TCGA) were retrieved from the UCSC Xena database (https://xena.ucsc.edu/). *ADAM9* gene expression levels were examined in relation to various clinical features, including Gleason scores, clinical stages, pathological tumor sizes, and lymph node involvement. The Wilcoxon signed-rank test was used for comparisons between two groups, while the Kruskal-Wallis test followed by Dunn's post-hoc test was applied for features with more than two groups.

### Statistical analysis

Between-group differences in demographic characteristics were analyzed using a Chi-square test and Student's *t*-tests. Multivariate logistic regression models were employed to estimate odds ratios (ORs) and adjusted ORs (AORs) with 95% confidence intervals (CIs) of associations between genotypic frequencies and clinicopathologic features. All analyses were conducted using SAS software (vers. 9.1, 2005, for Windows; SAS Institute, Cary, NC, USA), with statistical significance set at *p* < 0.05.

## Results

### Demographic characteristics of PCa patients

In Table 1, comparisons are presented of demographic characteristics of PCa patients with postoperative BCR (222 patients) to those without BCR (480 patients). Patients with BCR had a significantly higher incidence of advanced clinical T stages (T3+T4) and elevated PSA levels (> 10 ng/mL) at diagnosis compared to those without BCR. Surgical pathological findings also revealed that patients with BCR more frequently exhibited high pathologic Gleason grades (3+4+5), advanced pathologic T (T3+T4) and N (N1) stages, and evidence of seminal vesicle, perineural, and lymphovascular invasion by tumor cells. According to the D'Amico risk classification for PCa, it was observed that a higher proportion of patients with BCR fell into the high-risk group. Overall, demographic characteristics of our recruited PCa subjects, both with and without BCR, were consistent with those reported in previous studies 25.

### Associations of ADAM9 SNPs with postoperative BCR in PCa patients

We then explored the potential effects of four selected SNPs (rs6474526 (T/G), rs7006414 (T/C), rs10105311 (C/T), and rs78451751 (T/C)) in the *ADAM9* gene on BCR in PCa patients after an RP. Genotype frequencies of these SNPs were initially analyzed in our cohort of 702 PCa patients. The most common alleles were homozygous T/T for rs6474526, rs7006414, and rs78451751, while homozygous C/C was most prevalent for rs10105311 (Table 2). After adjusting for all potential confounders, only the ADAM9 rs7006414 T/C genotype showed a significant association with a higher risk of postoperative BCR (AOR = 1.508, 95% CI = 1.011-2.250) compared to the rs7006414 T/T genotype (Table 2). No significant trends were observed in polymorphic frequencies of rs6474526, rs10105311, or rs78451751.

In patients aged under 65 years, those carrying the ADAM9 rs7006414 polymorphism also had an increased risk of BCR under both the codominant model (TC vs. TT: AOR = 2.105, 95% CI = 1.111-3.988) and dominant model (TC + CC vs. TT: AOR = 1.432, 95% CI = 1.050-1.953) (Table 3). However, no associations were observed in elderly PCa patients (over 65 years) (data not shown). Additionally, our findings revealed that younger PCa patients carrying at least one minor allele of rs6474526 (TG + GG) had an increased risk of BCR (Table 3). These results provide insights into potential associations between *ADAM9* genetic variations and BCR, particularly in younger PCa patients after an RP.

### Relationships between clinicopathological features and ADAM9 SNPs in PCa patients with and those without BCR

Next, to clarify the effects of *ADAM9* genetic polymorphisms on the clinicopathological status of PCa, we examined factors such as pathologic staging, pathologic Gleason grade groups, clinical staging, tumor invasion statuses, and D'Amico classification. Among the four ADAM9 loci analyzed, we found that PCa patients carrying at least one minor allele (TG+GG) of rs6474526 had a significantly higher risk of developing advanced clinical T stages (cT3+4) (OR = 2.016, 95% CI = 1.101-3.690; *p* = 0.021) compared to those with the wild-type (WT) homozygote (TT), as shown in Table 4. None of the other three ADAM9 SNPs—rs7006414, rs10105311, or rs78451751—showed significant associations with the clinicopathological features mentioned above (see Table 4 and data not shown). We further stratified PCa patients into subgroups with or without BCR and investigated relationships between ADAM9 SNPs and PCa clinicopathological statuses within both groups. Notably, among the 480 PCa patients without BCR, those carrying the rs6474526 G-allele had a significantly higher risk of developing advanced clinical T stages (OR = 3.241, 95% CI = 1.433-7.328; *p* = 0.003) compared to patients with the WT T-allele (Table 5).

### Potential impacts of ADAM9 genetic polymorphisms on ADAM9 expression

**Figure 1:**
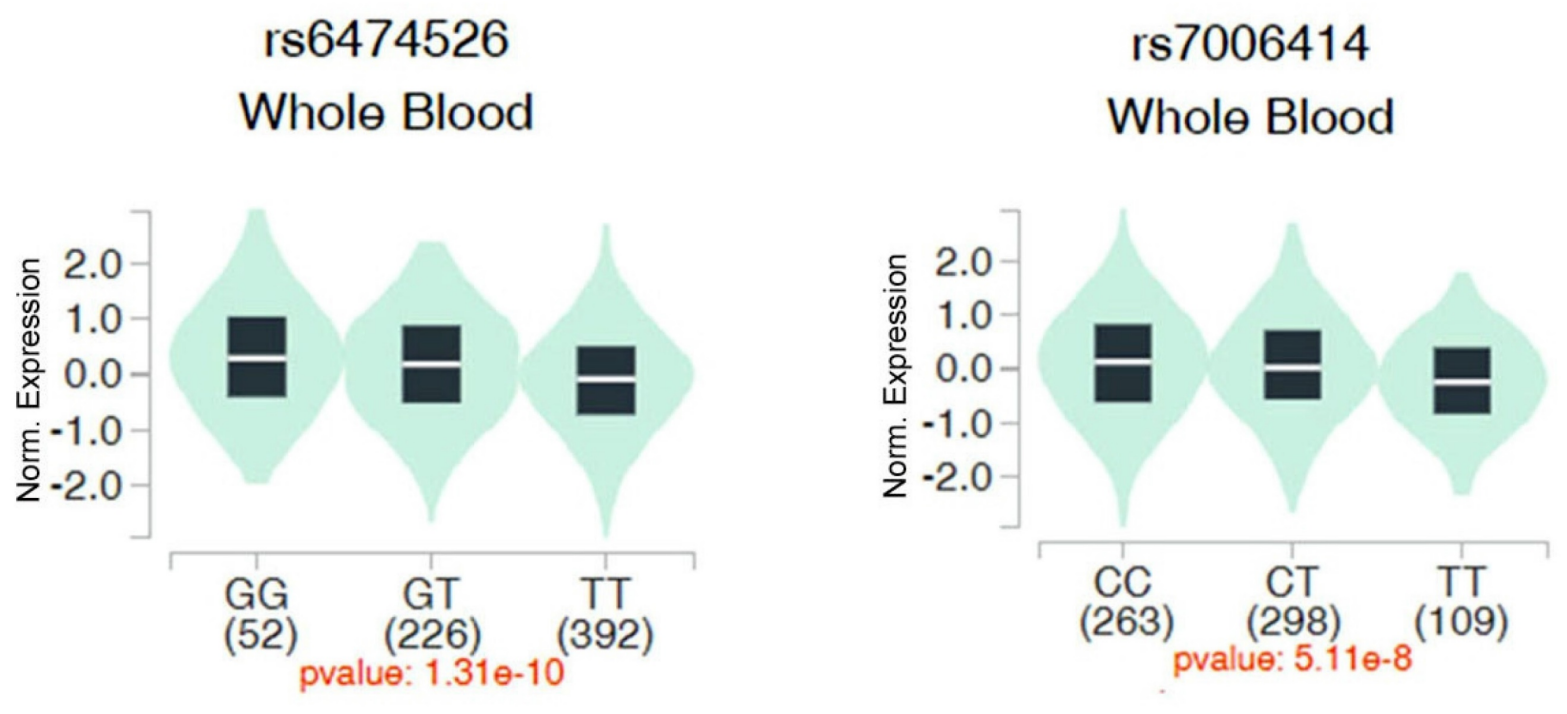
Impacts of ADAM9 rs6474526 and rs7006414 polymorphisms on ADAM9 expression were assessed using validated data from the Genotype Tissue Expression (GTEx) portal (https://www.gtexportal.org/home/). Violin plots illustrate that the G allele of rs6474526 (left panel) and the C allele of rs7006414 (right panel) were associated with higher ADAM9 expression levels in whole blood.

We then evaluated associations between ADAM9 polymorphisms and *ADAM9* gene expression in whole blood samples from healthy individuals using data from the Genotype-Tissue Expression (GTEx) database. Individuals with the WT homozygous TT genotype for rs6474526 and rs7006414 all showed the lowest ADAM9 expression levels compared to those carrying at least one minor allele (Figure 1).

### Correlations of ADAM9 expression levels with clinicopathologic features and prognoses of PCa patients

**Figure 2:**
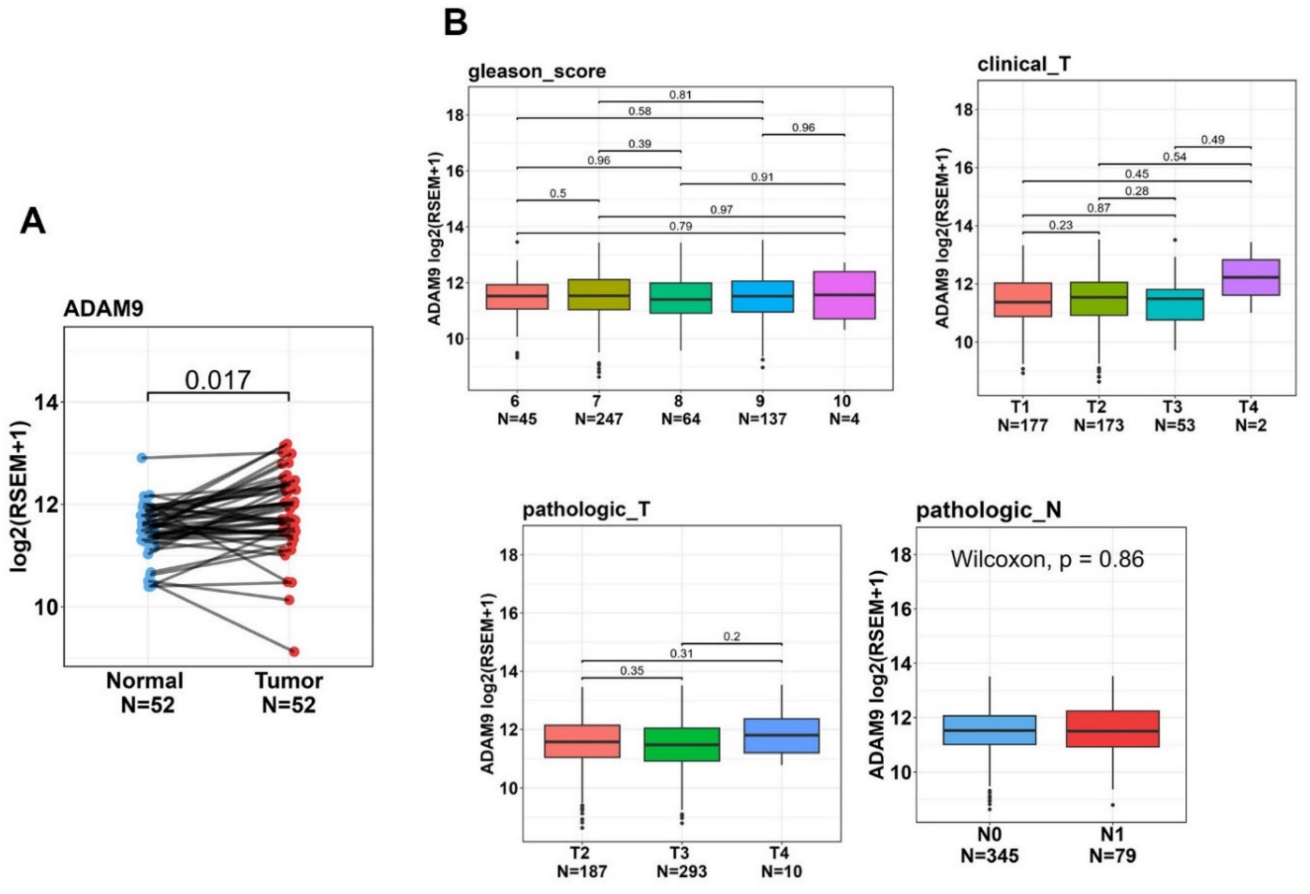
The clinical relevance of ADAM9 levels in prostate cancer (PCa) patients was analyzed using data from the TCGA-prostate adenocarcinoma (PRAD) dataset. (A) *ADAM9* gene expressions were evaluated in paired normal and tumor tissues from PCa patients. (B) *ADAM9* gene expression levels in PCa were compared from the TCGA-PRAD dataset based on Gleason scores, clinical T stages, pathological T stages, and lymph node metastasis.

To further examine ADAM9 expression levels in normal and PCa tissues and explore correlations between ADAM9 levels and disease progression, we utilized the TCGA-PRAD dataset. Our analysis revealed that ADAM9 expression was significantly higher in tumor tissues compared to corresponding matched normal tissues (Figure 2A). Although we found no significant correlations between elevated ADAM9 expression levels and various clinicopathological features, such as Gleason scores, clinical T stages, pathological T stages, and lymph node metastasis, we observed a trend where tumor tissues with a clinical T4 stage exhibited higher ADAM9 expression compared to those with clinical T1-T3 stages (Figure 2B).

## Discussion

Given the significant role of ADAM9 as a protease with oncogenic effects in PCa progression 17,18, we identified polymorphisms in the promoter and intron regions of the *ADAM9* gene that displayed different distributions in PCa patients with and those without BCR. Our findings showed that patients with the mutant TC genotype of rs7006414 had a significantly higher risk of developing postoperative BCR, with stronger associations observed in younger PCa patients (aged ≤ 65 years).

In this subgroup, we also found that those carrying at least one minor G allele of rs6474526 had an increased risk of BCR. These results highlight the potential influence of specific genetic variations of ADAM9 on BCR, particularly in younger PCa patients after an RP. Additionally, PCa patients with a mutated G variant of ADAM9 rs6474526 exhibited a notably increased risk of developing advanced clinical T stages (3 or 4). More-robust associations between the rs6474526 SNP and advanced clinical T stages were observed in PCa patients without BCR, suggesting that the ADAM9 rs6474526 SNP may influence tumor growth, especially in patients without BCR.

The rs7006414 SNP is located at position -1314 in the promoter region of the *ADAM9* gene. Results of an ADAM9 promoter reporter assay demonstrated that the rs7006414 C allele exhibited higher transcriptional activity compared to the rs7006414 T allele in both neural and non-neural cell lines, such as the LNCaP PCa cell line 26, thereby increasing ADAM9 expression. This difference in activity may be due to variations in the affinity of DNA-binding proteins for the two alleles of the ADAM9 promoter. The same study indicated that the rs7006414 C allele enhances DNA/protein interactions, as shown by electrophoretic mobility shift assay results, suggesting that the rs7006414 C polymorphism may more effectively bind with transcription factors, leading to increased ADAM9 expression 26. Consistent with these findings, we also observed elevated ADAM9 expression in whole blood tissues of individuals carrying the polymorphic C allele of ADAM9 rs7006414, as indicated by data from the GTEx database. Indeed, elevated ADAM9 levels were identified as a prognostic marker for BCR in PCa patients 20. Collectively, these observations suggest that the polymorphic C allele of ADAM9 rs7006414 may upregulate ADAM9 expression, thereby increasing the likelihood of BCR in PCa patients after an RP.

ADT is the primary treatment strategy for PCa as it reduces circulating androgen levels to castration levels 27. ADAM9 upregulation was reported to play a critical role in resistance to ADT in PCa patients 19. In addition to androgens, estrogens—particularly estradiol, the most potent endogenous estrogen—also influence PCa cell biology. For instance, plasma estradiol levels are a significant predictor of high-grade PCa in patients undergoing an RP 28 and in patients undergoing ADT who progress to CRPC 29. Cong *et al.* showed estrogen to be an inducer of ADAM9 promoter activity in LNCaP cells, especially in cases where the *ADAM9* gene carries the rs7006414 C polymorphism 20. We propose that age-related estrogen declines may lead to lower ADAM9 levels in older PCa patients, which could explain why we observed stronger associations between the rs7006414 SNP and BCR in younger PCa patients after an RP.

Beyond rs7006414, our findings indicated that the G polymorphism of ADAM9 rs6474526 also affected BCR and advanced clinical T stages in PCa patients. Notably, previous research demonstrated that elevated ADAM9 expression promoted tumor growth in PCa 21. The rs6474526 SNP resides within an intron, and data from the GTEx database revealed that individuals with a polymorphic G allele of rs6474526 showed higher ADAM9 expression in whole blood tissues. Although intronic polymorphisms generally do not alter protein sequences, emerging evidence suggests that these variations can cause splicing abnormalities, potentially affecting transcription and contributing to various human diseases, including cancers. For example, the rs12203592 intronic polymorphism in the interferon regulatory factor 4 (*IRF4*) gene was linked to a higher risk of acute lymphoblastic leukemia, where a C to T substitution increased *IRF4* gene expression by altering the binding affinity of the activator protein 2α transcription factor (TF) 30. Additionally, intronic regions often contain numerous cis-regulatory elements, such as TF-binding sites, enhancers, and silencers, which can either upregulate or downregulate gene expressions. For instance, three independent variants (rs2981578, rs35054928, and rs45631563) in fibroblast growth factor receptor 2 (FGFR2) were mapped to transcriptional silencer elements, enhancing silencer activity, reducing FGFR2 expression, and increasing breast cancer risk due to greater estrogen responsiveness 31. Moreover, intronic regions also encompass many non-coding RNA motifs, including long non-coding (lnc)RNAs. Intronic lncRNAs were shown to play crucial roles in regulating expressions of their host genes 32. Interestingly, lncADAM9 was reported to downregulate *ADAM9* mRNA 33. However, whether the rs6474526 SNP is located within lncADAM9 or another intronic lncRNA region remains unclear. The transcriptional potential of the rs6474526 T>G SNP within the ADAM9 intron will be further investigated in future studies.

Nevertheless, this study still has some limitations that need to be discussed. First, due to the relatively small sample size, the frequencies of certain homozygous variants (e.g., rs7006414 CC or rs6474526 GG genotypes) were low in subgroups, which may have limited the statistical power and precision of our results. Therefore, conducting studies with larger independent cohorts from different medical centers would provide more robust and reliable findings on the impact of ADAM9 SNPs on BCR and the development of PCa. Additionally, all PCa patients in this SNP study were Taiwanese (of Chinese ethnicity), whereas the correlations between ADAM9 expression and clinicopathologic features were analyzed using the TCGA-PRAD dataset, which predominantly includes White and African American individuals. It remains unclear whether similar results would be observed across different racial/ethnic groups. Further investigations of these SNPs in diverse racial populations of PCa patients are needed to confirm our findings. Finally, our current study only demonstrated the effects of ADAM9 rs6474526 and rs7006414 SNPs on ADAM9 gene expression in whole blood tissues of healthy individuals, based on the GTEx database. To validate the influence of ADAM9 SNPs on ADAM9 expression in PCa patients, future studies should simultaneously collect mRNA and DNA from the same samples of PCa patients.

In summary, ours is the first study to explore distinct allelic effects of ADAM9 SNPs (rs7006414 and rs6474526) in a Taiwanese population, highlighting their impacts on the incidence of BCR and tumor growth in PCa. Our findings suggest that the rs7006414 promoter SNP and the rs6474526 intronic SNP may influence *ADAM9* gene expression, thereby contributing to promoting BCR and tumor growth in PCa. These genetic variants could potentially serve as valuable markers for predicting recurrence in PCa patients undergoing an RP.

## Figures and Tables

**Table 1:** Distributions of demographic characteristics among 702 prostate cancer patients

Variable	Biochemical recurrence (BCR)		
	No (*N*=480)	Yes (*N*=222)	*p* value
Age at diagnosis (years)			
≤ 65	206 (42.9%)	91 (41.0%)	0.631
> 65	274 (57.1%)	131 (59.0%)	
PSA at diagnosis (ng/mL)			
≤ 10	267 (55.6%)	67 (30.2%)	< 0.001*
> 10	213 (44.4%)	155 (69.8%)	
Pathologic Gleason grade group			
1+2	351 (73.1%)	70 (31.5%)	< 0.001*
3+4+5	129 (26.9%)	152 (68.5%)	
Clinical T (tumor) stage			
1+2	438 (91.2%)	167 (75.2%)	< 0.001*
3+4	42 (8.8%)	55 (24.8%)	
Clinical N (node) stage			
N0	473 (98.5%)	215 (96.8%)	0.135
N1	7 (1.5%)	7 (3.2%)	
Pathologic T stage			
2	319 (66.5%)	52 (23.4%)	< 0.001*
3+4	161 (33.5%)	170 (76.6%)	
Pathologic N stage			
N0	468 (97.5%)	174 (78.4%)	< 0.001*
N1	12 (2.5%)	48 (21.6%)	
Seminal vesicle invasion			
No	435 (90.6%)	117 (52.7%)	< 0.001*
Yes	45 (9.4%)	105 (47.3%)	
Perineural invasion			
No	168 (35.0%)	18 (8.1%)	< 0.001*
Yes	312 (65.0%)	204 (91.9%)	
Lymphovascular invasion			
No	446 (92.9%)	144 (64.9%)	< 0.001*
Yes	34 (7.1%)	78 (35.1%)	
D'Amico classification			
Low risk/Intermediate risk	273 (56.9%)	75 (33.8%)	< 0.001*
High risk	207 (43.1%)	147 (66.2%)	

* A *p* value of < 0.05 indicates statistical significance.

**Table 2:** Distribution frequencies of ADAM9 genotypes in 702 prostate cancer patients with or without biochemical recurrence (BCR)

Variable	BCR			
	No (*N*=480)	Yes (*N*=222)	AOR (95% CI)	*p* value
rs6474526				
TT	437 (91.0%)	195 (87.8%)	1.000	
TG	41 (8.5%)	26 (11.7%)	1.620 (0.866~3.032)	0.131
GG	2 (0.5%)	1 (0.5%)	0.827 (0.022~30.603)	0.918
TG+GG	43 (9.0%)	27 (12.2%)	1.262 (0.925~1.720)	0.142
rs7006414				
TT	276 (57.5%)	120 (54.1%)	1.000	
TC	171 (35.6%)	93 (41.9%)	1.508 (1.011~2.250)	0.044*
CC	33 (6.9%)	9 (4.0%)	0.738 (0.283~1.927)	0.535
TC+CC	204 (42.5%)	102 (45.9%)	1.181 (0.973~1.434)	0.092
rs10105311				
CC	309 (64.4%)	143 (64.4%)	1.000	
CT	151 (31.5%)	72 (32.4%)	1.230 (0.815~1.856)	0.324
TT	20 (4.1%)	7 (3.2%)	0.804 (0.256~2.525)	0.709
CT+TT	171 (35.6%)	79 (35.6%)	1.088 (0.891~1.329)	0.406
rs78451751				
TT	473 (98.5%)	220 (99.1%)	1.000	
TC	7 (1.5%)	2 (0.9%)	0.539 (0.183~1.589)	0.263
CC	0 (0.0%)	0 (0.0%)	---	---
TC+CC	7 (1.5%)	2 (0.9%)	0.539 (0.183~1.589)	0.263

Odds ratios (ORs) and with their 95% confidence intervals (CIs) were estimated by logistic regression models. Adjusted ORs (AORs) with their 95% CIs were estimated by multiple logistic regression models after controlling for age at diagnosis, prostate-specific antigen, pathologic Gleason grade group, clinical T stage, clinical N stage, clinical M stage, pathologic T stage, seminal vesicle invasion, perineural invasion, lymphovascular invasion, and D'Amico classification. * A *p* value of < 0.05 indicates statistical significance.

**Table 3:** Distribution frequencies of ADAM9 genotypes in 297 younger prostate cancer patients (aged ≤ 65 years) with or without biochemical recurrence (BCR)

Variable	BCR			
	No (*N*=206)	Yes (N=91)	AOR (95% CI)	*p* value
rs6474526				
TT	193 (93.7%)	78 (85.7%)	1.000	
TG	13 (6.3%)	13 (14.3%)	1.769 (1.085~2.884)	0.022*
GG	0 (0.0%)	0 (0.0%)	---	---
TG+GG	13 (6.3%)	13 (14.3%)	1.769 (1.085~2.884)	0.022*
rs7006414				
TT	117 (56.8%)	47 (51.6%)	1.000	
TC	75 (36.4%)	39 (42.9%)	2.105 (1.111~3.988)	0.025*
CC	14 (6.8%)	5 (5.5%)	1.680 (0.432~6.529)	0.454
TC+CC	89 (43.2%)	44 (48.4%)	1.432 (1.050~1.953)	0.023*
rs10105311				
CC	128 (62.1%)	60 (65.9%)	1.000	
CT	67 (32.5%)	26 (28.6%)	1.221 (0.636~2.343)	0.549
TT	11 (5.3%)	5 (5.5%)	1.767 (0.433~7.207)	0.428
CT+TT	78 (37.9%)	31 (34.1%)	1.131 (0.829~1.544)	0.437
rs78451751				
TT	202 (98.1%)	91 (100.0%)	1.000	
TC	4 (1.9%)	0 (0.0%)	---	---
CC	0 (0.0%)	0 (0.0%)	---	---
TC+CC	4 (1.9%)	0 (0.0%)	---	---

Odds ratios (ORs) with their 95% confidence intervals (CIs) were estimated by logistic regression models. Adjusted odds ratios (AORs) with their 95% CIs were estimated by multiple logistic regression models after controlling for prostate-specific antigen, pathologic Gleason grade group, clinical T stage, clinical N stage, pathologic T stage, pathologic N stage, seminal vesicle invasion, perineural invasion, lymphovascular invasion, and D'Amico classification. * A *p* value of < 0.05 indicates statistical significance.

**Table 4:** Odds ratios (ORs) and 95% confidence intervals (CIs) for the clinical status and genotypic frequencies of ADAM9 rs6474526 and rs7006414 in 702 prostate cancer patients

Variable	rs6474526				rs7006414			
	TT (*N*=632)	TG+GG (*N*=70)	OR (95% CI)	*p* value	TT (N=396)	TC+CC (N=306)	OR (95% CI)	*p* value
Pathologic Gleason grade group								
1+2	380 (60.1%)	41 (58.6%)	1.000	0.801	235 (59.3%)	186 (60.8%)	1.000	0.699
3+4+5	252 (39.9%)	29 (41.4%)	1.067 (0.646~1.761)		161 (40.7%)	120 (39.2%)	0.942 (0.694~1.277)	
Clinical T (tumor) stage								
1+2	551 (87.2%)	54 (77.1%)	1.000	0.021*	343 (86.6%)	262 (85.6%)	1.000	0.705
3+4	81 (12.8%)	16 (22.9%)	2.016 (1.101~3.690)		53 (13.4%)	44 (14.4%)	1.087 (0.706~1.672)	
Clinical N (node) stage								
N0	619 (97.9%)	69 (98.6%)	1.000	0.721	387 (97.7%)	301 (98.4%)	1.000	0.548
N1	13 (2.1%)	1 (1.4%)	0.690 (0.089~5.356)		9 (2.3%)	5 (1.6%)	0.714 (0.237~2.153)	
Pathologic T stage								
2	338 (53.5%)	33 (47.1%)	1.000	0.313	205 (51.8%)	166 (54.2%)	1.000	0.514
3+4	294 (46.5%)	37 (52.9%)	1.289 (0.786~2.114)		191 (48.2%)	140 (45.8%)	0.905 (0.671~1.221)	
Pathologic N stage								
N0	580 (91.8%)	62 (88.6%)	1.000	0.363	361 (91.2%)	281 (91.8%)	1.000	0.753
N1	52 (8.2%)	8 (11.4%)	1.439 (0.654~3.168)		35 (8.8%)	25 (8.2%)	0.918 (0.537~1.569)	
Seminal vesicle invasion								
No	500 (79.1%)	52 (74.3%)	1.000	0.350	308 (77.8%)	244 (79.7%)	1.000	0.530
Yes	132 (20.9%)	18 (25.7%)	1.311 (0.742~2.317)		88 (22.2%)	62 (20.3%)	0.889 (0.617~1.282)	
Perineural invasion								
No	171 (27.1%)	15 (21.4%)	1.000	0.311	104 (26.4%)	82 (26.8%)	1.000	0.874
Yes	461 (72.9%)	55 (78.6%)	1.360 (0.748~2.471)		292 (73.7%)	224 (73.2%)	0.973 (0.694~1.364)	
Lymphovascular invasion								
No	529 (83.7%)	61 (87.1%)	1.000	0.456	334 (84.3%)	256 (83.7%)	1.000	0.806
Yes	103 (16.3%)	9 (12.9%)	0.758 (0.365~1.574)		62 (15.7%)	50 (16.3%)	1.052 (0.701~1.580)	
D'Amico classification								
Low risk/Intermediate risk	312 (49.4%)	36 (51.4%)	1.000	0.743	185 (46.7%)	163 (53.3%)	1.000	0.085
High risk	320 (50.6%)	34 (48.6%)	0.921 (0.562~1.509)		211 (53.3%)	143 (46.7%)	0.769 (0.570~1.037)	

ORs with their 95% CIs were estimated by logistic regression models. * *p* < 0.05 indicates statistical significance.

**Table 5:** Odds ratios (ORs) and 95% confidence intervals (CIs) for the clinical status and ADAM9 rs6474526 genotypic frequencies in 702 prostate cancer patients with and those without biochemical recurrence

Variable	No biochemical recurrence (*N*=480)				With biochemical recurrence (*N*=222)			
	TT (*N*=437)	TG+GG (*N*=43)	OR (95% CI)	*p* value	TT (*N*=195)	TG+GG (*N*=27)	OR (95% CI)	*p* value
Pathologic Gleason grade group								
1+2	319 (73.0%)	32 (74.4%)	1.000	0.841	61 (31.3%)	9 (33.3%)	1.000	0.830
3+4+5	118 (27.0%)	11 (25.6%)	0.929 (0.454~1.903)		134 (68.7%)	18 (66.7%)	0.91 (0.387~2.142)	
Clinical T (tumor) stage								
1+2	404 (92.4%)	34 (79.1%)	1.000	0.003*	147 (75.4%)	20 (74.1%)	1.000	0.882
3+4	33 (7.6%)	9 (20.9%)	3.241 (1.433~7.328)		48 (24.6%)	7 (25.9%)	1.072 (0.427~2.691)	
Clinical N (node) stage								
N0	431 (98.6%)	69 (97.7%)	1.000	0.619	188 (96.4%)	27 (100.0%)	1.000	---
N1	6 (1.4%)	1 (2.3%)	1.710 (0.201~14.545)		7 (3.6%)	0 (0.0%)	---	
Pathologic T stage								
2	292 (66.8%)	27 (62.8%)	1.000	0.593	46 (23.6%)	6 (22.2%)	1.000	0.875
3+4	145 (33.2%)	16 (37.2%)	1.193 (0.623~2.285)		149 (76.4%)	21 (77.8%)	1.081 (0.411~2.838)	
Pathologic N stage								
N0	428 (97.9%)	40 (93.0%)	1.000	0.083	152 (77.9%)	22 (81.5%)	1.000	0.676
N1	9 (2.1%)	3 (7.0%)	3.567 (0.928~13.706)		43 (22.1%)	5 (18.5%)	0.803 (0.287~2.247)	
Seminal vesicle invasion								
No	398 (91.1%)	37 (86.0%)	1.000	0.280	102 (52.3%)	15 (56.6%)	1.000	0.751
Yes	39 (8.9%)	6 (14.0%)	1.655 (0.657~4.166)		93 (47.7%)	12 (44.4%)	0.877 (0.391~1.971)	
Perineural invasion								
No	156 (35.7%)	12 (27.9%)	1.000	0.307	15 (7.7%)	3 (11.1%)	1.000	0.542
Yes	281 (64.3%)	31 (72.1%)	1.434 (0.716~2.872)		180 (92.3%)	24 (88.9%)	0.667 (0.180~2.473)	
Lymphovascular invasion								
No	405 (92.7%)	41 (95.3%)	1.000	0.515	124 (63.6%)	20 (74.1%)	1.000	0.285
Yes	32 (7.3%)	2 (4.7%)	0.617 (0.143~2.670)		71 (36.4%)	7 (25.9%)	0.611 (0.246~1.517)	
D'Amico classification								
Low risk/Intermediate risk	248 (56.8%)	25 (58.1%)	1.000	0.861	64 (32.8%)	11 (40.7%)	1.000	0.415
High risk	189 (43.2%)	18 (41.9%)	0.945 (0.501~1.782)		131 (67.2%)	16 (59.3%)	0.711 (0.312~1.620)	

ORs with their 95% CIs were estimated by logistic regression models. * *p* < 0.05 indicates statistical significance.
